# Serology for Trachoma Surveillance after Cessation of Mass Drug Administration

**DOI:** 10.1371/journal.pntd.0003555

**Published:** 2015-02-25

**Authors:** Diana L. Martin, Rhiannon Bid, Frank Sandi, E. Brook Goodhew, Patrick A. Massae, Augustin Lasway, Heiko Philippin, William Makupa, Sandra Molina, Martin J. Holland, David C. W. Mabey, Chris Drakeley, Patrick J. Lammie, Anthony W. Solomon

**Affiliations:** 1 Division of Parasitic Diseases and Malaria, Centers for Disease Control and Prevention, Atlanta, Georgia, United States of America; 2 Clinical Research Department, Faculty of Infectious and Tropical Diseases, London School of Hygiene and Tropical Medicine, London, United Kingdom; 3 Kilimanjaro Christian Medical University College, Moshi, Tanzania; 4 The University of Dodoma, Dodoma, Tanzania; 5 Department of Ophthalmology, Kilimanjaro Christian Medical Centre, Moshi, Tanzania; 6 Huruma Hospital, Mkuu, Tanzania; 7 Department of Immunology and Infection, Faculty of Infectious and Tropical Diseases, London School of Hygiene and Tropical Medicine, London, United Kingdom; University of California San Francisco, UNITED STATES

## Abstract

**Background:**

Trachoma, caused by *Chlamydia trachomatis (Ct)*, is the leading infectious cause of blindness worldwide. Yearly azithromycin mass drug administration (MDA) plays a central role in efforts to eliminate blinding trachoma as a public health problem. Programmatic decision-making is currently based on the prevalence of the clinical sign “trachomatous inflammation-follicular” (TF) in children. We sought to test alternative tools for trachoma surveillance based on serology in the 12-year cohort of Kahe Mpya, Rombo District, Tanzania, where ocular chlamydial infection was eliminated with azithromycin MDA by 2005.

**Methodology and Principal Findings:**

The present study was a community-based cross-sectional survey in Kahe Mpya. Of 989 residents, 571 people aged 6 months to 87 years were enrolled: 58% of the total population and 73% of 1–9 year olds, the key WHO indicator age group. Participants were examined for TF, had conjunctival swabs collected for nucleic acid amplification test (NAAT)-based detection of *Ct*, and blood collected for analysis of antibodies to the *Ct* antigens pgp3 and CT694 by multiplex bead-based immunoassay. Seroconversion rate was used to estimate changes in the force of infection in a reversible catalytic model. No conjunctival swabs tested positive for *Ct* infection by NAAT. Among 1–9 year olds, TF prevalence was 6.5%, whereas only 3.5% were seropositive. Force of infection modelling indicated a 10-fold decrease in seroconversion rate at a time corresponding to MDA commencement. Without baseline serological data, the inferences we can make about antibody status before MDA and the longevity of the antibody response are limited, though our use of catalytic modelling overcomes some of these limitations.

**Conclusions/Significance:**

Serologic tests support NAAT findings of very low to zero prevalence of ocular *Ct* in this community and have potential to provide objective measures of transmission and useful surveillance tools for trachoma elimination programs.

## Author Summary

Trachoma is the leading infectious cause of blindness. The infectious agent, *Chlamydia trachomatis*, can be treated with a single oral dose of azithromycin. Donated drug is a cornerstone of programs dedicated to the elimination of trachoma as a public health problem. Azithromycin is given to the entire district for 3–5 years when 10% or more of 1–9 year-olds in the district have signs of a defined follicular conjunctivitis in one or both eyes. However, follicles can be difficult to reliably diagnose and can be caused by other pathogens, especially in settings with low trachoma prevalence. More sensitive and specific ways to assess communities for trachoma transmission at program endpoints are needed. Herein we examined antibody responses in children living in a community in Tanzania born after stopping drug treatment 10 years previously. Low antibody levels (3.5% in 1–9 year-olds) reflected the lack of ocular chlamydial infection in these children. We also modelled the data to show that changes in age-specific antibody prevalence occurred when the mass drug treatment stopped. These data suggest that the age-specific prevalence of antibody responses may be of use to programs seeking to demonstrate the impact of interventions against trachoma.

## Introduction

Trachoma, caused by the bacterium *Chlamydia trachomatis (Ct)*, is the leading infectious cause of blindness worldwide [1]. Infection can manifest clinically in a number of ways, including follicular conjunctivitis, classified as “trachomatous inflammation-follicular” (TF) in the WHO simplified grading system [2] if five or more follicles are present in the central upper tarsal conjunctiva; and/or inflammatory thickening, classified as “trachomatous inflammation-intense” (TI) if more than half of the deep tarsal vessels are obscured. Repeated infections can lead to conjunctival scarring (TS) and trichiasis (TT), in which in-turned eyelashes rub against the globe and may result in visual impairment or blindness caused by corneal opacity (CO) [3]. Azithromycin mass drug administration (MDA), recommended where the prevalence of TF is ≥10% in children aged 1–9 years, is a critical component of the strategy for Global Elimination of Trachoma by 2020 (GET2020) [4]. The current WHO endpoint for cessation of community-based antibiotic treatment is a TF prevalence in 1–9 year-olds of <5%.

Prevalence surveys illustrate that signs of active trachoma, TF and TI, exceed *Ct* infection rates. Follicular or intense conjunctivitis may be caused by non-chlamydial bacteria, with the relative importance of this phenomenon probably increasing after populations begin to receive azithromycin MDA [5]. Furthermore, the examination process can be difficult to standardize [6–9]; inter-observer agreement is often sub-optimal. The poor correspondence between signs and infection—seen at both individual and community level—is problematic, given that field grading is the basis of public health decision-making [5, 10].

As trachoma elimination efforts are intensified globally and interventions move populations towards trachoma elimination goals, the availability of a post-elimination surveillance methodology with greater reliability than clinical examination will become increasingly important to allow programs to identify and respond to recrudescent infection. Recent efforts to evaluate serology as a viable option for post-MDA surveillance identified tests using two previously-described chlamydial antigens, pgp3 and CT694, as having high sensitivity to detect current ocular infection, and high specificity using non-endemic controls [11]. The age-specific prevalence of serological responses to *Ct* antigens at community level could provide an informative proxy measure of intensity of transmission and an early indicator of transmission recrudescence. This study therefore examined the use of serological tools for monitoring and evaluation in a post-MDA setting by assessing the age-specific prevalence of signs of trachoma and *Ct*-specific antibody responses within a community in which MDA ceased in 2002 and ocular *Ct* infection was subsequently found to have been eliminated in 2005 [12].

## Materials and Methods

### Study area

This study was conducted in the Tanzanian community of Kahe Mpya, Rombo District. Kahe Mpya consists of approximately 250 households, with a population (in July 2012) of 989. A Kilimanjaro Christian Medical College (KCMC)/London School of Hygiene & Tropical Medicine (LSHTM)/Huruma Hospital collaboration has been conducting trachoma research in this community since 2000 [13, 14]. High coverage azithromycin MDA was delivered in 2000 and 2002, and topical tetracycline ointment treatment was given, at intervals between 2000 and 2005, to individuals with active trachoma; elimination of ocular *Ct* infection by 2005 was previously documented [12]. Ethical approval to carry out this research was obtained from the ethics committees at LSHTM (UK), Centers for Disease Control and Prevention (USA), KCMC / Tumaini University, and the National Institute for Medical Research (TZ). All adults provided written informed consent, and for children under 18, the consent of a parent or guardian was obtained.

All Kahe Mpya residents were invited by village leaders to a series of central locations, where those consenting to the study underwent examination of both eyes by a trained, highly experienced ophthalmic nurse known to the community, using binocular loupes (magnification ×2·5) and a torch. Signs of trachoma were graded according to the WHO simplified grading system [2]. After examination, swabs were collected from the everted upper eyelid of the right eye using a sterile polyester-tipped-swab by passing the swab across the conjunctiva four times. Swabs were placed into sterile polypropylene tubes and kept at 4°C until frozen (-20°C). Individuals with signs of active trachoma were given a tube of 1% tetracycline eye ointment free of charge and instructed to apply it daily to both eyes for six weeks. Fingerprick blood was collected by Tanzanian registered physicians onto filter paper with six circular extensions, calibrated so that each extension absorbed 10μl of whole blood (TropBio Pty Ltd, Townsville, Queensland, Australia). Each filter paper was air-dried then individually placed in a zip-lock bag and frozen (-20°C). Each sample was affixed with a pre-printed bar-coded label that linked all samples from an individual but had no other patient identifier.

### Multiplex analysis for serum IgG

Dried blood spots were shipped to the Centers for Disease Control and Prevention in Atlanta GA, USA, for detection of IgG antibodies against the previously described chlamydial proteins pgp3 and CT694, on the Luminex platform, using previously defined cut-offs for positivity [11]. Briefly, serum eluted from dried blood spots was incubated with microbeads coupled to the antigens of interest, then excess serum washed off and bound antibody detected with an anti-human IgG and anti-human IgG4 biotinylated detection antibody, and finally detected using streptavidin-conjugated to phycoerythrin (PE). The fluorescent signal emitted by bound PE was converted to a median fluorescence intensity (MFI) with background from the blank subtracted out (MFI-BG). For pgp3, a MFI-BG value of 1024 was established as the low-limit value for positivity, with an indeterminate range of 1024 to 5998. For CT694, a MFI-BG value of 232 was established as the low-limit value for positivity, with an indeterminate range of 232 to 1982 [11].

### Force of infection modelling

To examine the change in transmission following MDA, we used seroconversion rate (SCR) to estimate the force of infection by fitting a simple reversible catalytic model to the measured seroprevalence, stratified into yearly age-groups, using maximum likelihood methods [15]. For these models only individuals aged one year and over were included to remove the effect of maternally derived antibodies in infants. Evidence for temporal changes in SCR was explored by fitting models in which the SCR was allowed to change at a single time-point. The significance of the change was identified using likelihood ratio tests against models with no change, and profile likelihoods were plotted to determine confidence intervals for the estimated time of the change.

### Detection of conjunctival swab *C*. *trachomatis* DNA

Samples were processed at the LSHTM and tested in pools of five using the Roche CT/NG Amplicor kit (Roche Molecular Systems, Pleasanton, CA, USA), with the intention of re-testing positive pools as individual samples [16–18]. Manufacturer’s instructions were followed except for sample extraction where a previously published protocol was used[14]. Two *Ct* positive and two *Ct* negative processing controls were run with each batch of specimens. According to the manufacturer’s directions, the Amplicor test was positive if the optical density read at 450 nm was ≥0·8, negative if the signal was <0·2, and equivocal if in-between. All equivocal tests were re-tested in duplicate, and only graded positive if at least one test was positive.

### Data entry and statistical analysis

All samples were analysed in anonymous fashion through the use of non-sequential sample codes linked only to patient records through the data collection sheet. Statistical analysis was carried out using STATA 12 and GraphPad Prism (version 6.0).

## Results

### Demographic information

The population and study population structure of Kahe Mpya sub-village is summarized in Table 1, based on census data collected in July 2012 for this study. From the total 989 residents of Kahe Mpya sub-village, 575 (58.1% coverage) people aged 0.2–87.6 years (median age 12.6, Table 1) participated in the study.

**Table 1:** Population structure of Kahe-Mpya and the study population.

**Kahe Mpya sub-village**	
Total population	989
Males [%]	481 [48.6]
Females [%]	508 [51.4]
**Population structure of study participants**	
Total number of participants	575
Males [%]	242 [42.1]
Females [%]	333 [57.9]
Age range in years [median]	0.2–87.6 [12.6]

### Age-specific prevalence of clinical signs of trachoma

The overall prevalence of active trachoma (TF,TI or both) in the examined population (n = 571; four individuals refused clinical exams) was 4.6%, with 21.5% exhibiting signs of scarring trachoma (TS/TT/CO, Table 2). There were no WHO simplified grading scheme signs of trachoma in 76·6% of the study group. The prevalence of TF amongst the WHO index age group (ages 1–9 years) was 6·5% (Table 2). Only one individual ≥ 10 years had TF. TS was absent in those <10 years, but was observed in all age groups >10 years (Table 2). TT was present only in individuals >20 years of age, with an overall population prevalence of 1% (Table 2). CO was only diagnosed in 2 individuals (0·4% of study participants), both of whom were over 70 years old (Table 2).

**Table 2:** Age-specific prevalence of clinical signs of trachoma.

Age (years)	TF [%]	TI [%]	TS [%]	TT [%]	CO [%]	N
<1	0	0	0	0	0	18
1	3 [20]	1 [7]	0	0	0	15
2	2 [8]	1 [4]	0	0	0	24
3	4 [14]	0	0	0	0	29
4	0	0	0	0	0	26
5	1 [6]	0	0	0	0	18
6	0	0	0	0	0	23
7	2 [8]	1 [4]	0	0	0	25
8	1 [9]	0	0	0	0	11
9	0	0	0	0	0	29
10–20	0	2[1]	8[5]	0	0	161
20–30	1 [4]	1 [4]	6 [25]	1 [4]	0	24
30–40	0	0	7 [19]	1 [3]	0	36
40–50	0	0	19 [61]	0	0	31
50–60	0	1 [2]	34 [74]	1 [2]	0	46
60–70	0	2 [6]	19 [58]	1 [3]	0	33
70–80	0	2 [12]	16 [94]	2 [12]	1 [6]	17
80–90	0	1 [20]	5 [100]	0	1 [20]	5
**TOTAL**	**14 [2.5]**	**12 [2.1]**	**114 [20.0]**	**6 [1.1]**	**2 [0.4]**	**571**
**TOTAL (0–9)**	**13 [6.0]**	**3 [1.4]**	**0**	**0**	**0**	**218**
**TOTAL (1–9)**	**13 [6.5]**	**3 [1.5]**	**0**	**0**	**0**	**200**

TF = trachomatous inflammation—follicular

TI = trachomatous inflammation—intense

TS = trachomatous scarring

TT = trachomatous trichiasis

CO = corneal opacity.

Numbers represent N for each group, numbers in parentheses represent % of individuals in each age group with the respective clinical sign.

### Age-specific seroprevalence of trachoma and *Ct* infection

**Fig 1:**
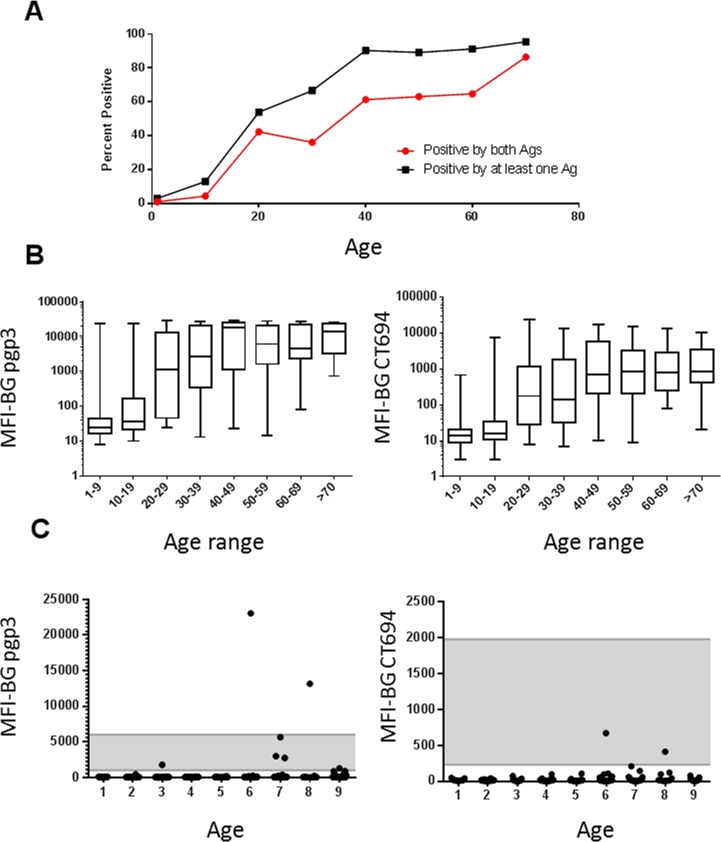
Antibody responses to *Ct* antigens 10 years after MDA cessation. A. Age-prevalence curves for antibody responses grouped by decade. Black squares represent individuals with any antibody-positive test (to pgp3 alone, CT694 alone, or both antigens), and red squares represent responses positive to both pgp3 and CT694. B. Plots show box-and-whiskers graph (min-max) of MFI-BG against age ranges grouped by decade for antibodies against pgp3 (left) and CT694 (right). C. Plots show age against MFI-BG for children aged 1–9. Each dot represents a single individual. Note the differences in the y-axis scales for pgp3 (left) and CT694 (right). Indeterminate range is shaded. Horizontal lines indicate cutoffs for antibody positivity. Ag = antigen.

Overall, 33.8% of participants were seropositive against at least one antigen (Fig. 1A). Seropositivity increased with age. By age 40, over 90% of participants tested positive to at least one antigen (pgp3 alone, CT694 alone, or both pgp3 and CT694, black squares, Fig. 1A), and over 60% tested positive to both antigens (Fig. 1A, red squares); this trend continued to the oldest age groups (Fig. 1A). The MFI also increased with age (Fig. 1B). Of 200 children aged 1–9, seven (3.5%) had antibody responses to one antigen, whereas only two (1%) had antibody responses to both antigens (Fig. 1C). Five of the seven samples with pgp3 reactivity fell into the indeterminate range, as did both of the CT694-reactive samples (Fig. 1C). Samples from six of the seven 1–9 year olds testing positive by serology were re-tested with separate pgp3 and CT694 bead sets and data replicated the original results (S1 Table).

None of the ocular swabs tested positive by NAAT.

### Force of infection modelling

**Fig 2:**
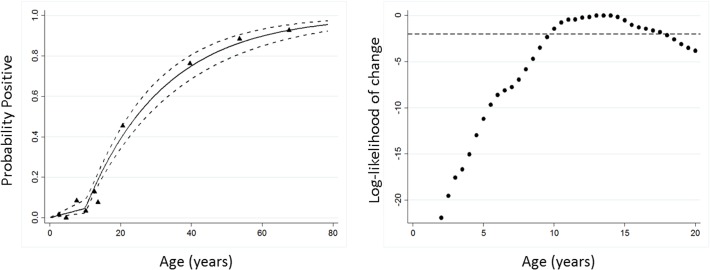
Force of infection modelling of seroconversion rates before and after MDA. A. Maximum likelihood fits from reversible catalytic equilibrium model for antibody responses either pgp3 or CT694 is shown. X-axis represents the time in years that each model has a change point. The y-axis is the log-likelihoods from each model where log-likelihoods are rescaled against a maximum of 0 and a log-likelihood above -2 is an approximate 95% confidence interval when the change occurred. B. A model in which SCR changed 10 years previously, to represent the time at which MDA ceased, had a better fit than the model that assumed the SCR had remained constant (likelihood ratio test X^2^ = 45.4 p,0.0001). The triangles represent deciles of observed seroprevalence; the solid blue line represents the predicted values based on the model with dotted lines and the 95% CI.

When a seroconversion model, which allowed for a single change in SCR, was fitted to the data, the best fit was provided by a change in transmission between 10–15 years previously, consistent with the timing of MDA in the years 2000 and 2002 (Fig. 2A for antibody responses to either antigen; responses to individual antigens gave similar profiles). We chose a model in which SCR changed 10 years previously, which had a better fit than the model that assumed the SCR had remained constant (Fig. 2B). The change in SCR before and after this change point is approximately a 10-fold reduction, from a pre-MDA SCR of 0.0448 (95%CI 0.0373–0.0537) to a post-MDA SCR of 0.004 (95%CI 0.0024–0.0093)].

## Discussion

Global efforts toward the elimination of blinding trachoma are being rapidly intensified, thanks to strong donor interest. As programs reduce the prevalence of disease and infection, robust surveillance systems will become crucial to detect any recrudescence in populations living in post-elimination settings. In this study, we examined the use of serological tools for trachoma in a post-MDA setting. The virtual absence of antibody responses in children born after MDA-precipitated elimination of ocular *Ct* infection reflects the lack of *Ct* transmission (as suggested by NAAT) and provides the first evidence that serological monitoring of antibody responses could be viable for informing programmatic decisions in the surveillance phase. Force of infection modelling shown here strongly supports the hypothesis that reductions in transmission in this community coincident with the commencement of azithromycin MDA were reflected in changes in *Ct* seroconversion rate. This suggests that serology could have a very useful programmatic role even in the absence of complete transmission interruption.

Several factors could contribute to the presence of signs of active trachoma in a community with low or no transmission of conjunctival *Ct*. First, the WHO simplified grading system employs strict criteria for diagnosis, but was designed for simplicity rather than specificity. Our grader was, however, well trained, highly experienced, and internationally certified, and we are confident of the accuracy of his judgements about the presence or absence of TF. Second, the natural histories of infection and disease differ, with signs arising weeks after infection has been acquired and persisting for weeks or months after infection clears. At the population level, the prevalence of infection declines more rapidly than the prevalence of TF following MDA [12, 19, 20] with some studies showing that TF persists at levels >10% within the population for months or years after infection has subsided [21, 22]. Finally, evidence suggests that, in low-trachoma-prevalence settings, the majority of TF is associated with conjunctival infection with non-chlamydial bacteria, including *S*. *pneumoniae* and *H*. *influenzae* [5, 23]. Non-bacterial causes of conjunctivitis such as adenovirus [24] may also contribute to TF clinical diagnoses in low-trachoma-prevalence settings. The use of photographs to validate field exams is becoming increasingly common but we have not found it to be reliable [25] and did not incorporate it into this study.

Antibodies against the *Ct* antigens among 1–9 years old in this study were present at very low prevalence and in general at very low densities. This is in stark contrast to areas of active transmission in which seropositivity exceeds rates of clinical disease, as would be expected from long-lived antibody responses.[26] and has high sensitivity for ocular infection,[11, 26] In the present study, antibody responses in 1–9 year olds may be *Ct*-specific, resulting from ocular or respiratory *Ct* infection acquired at birth from a mother with genital tract infection [27], or from ocular infection acquired outside the village or in the village itself. Because the target for trachoma programs is not the complete interruption of transmission, it would not be an indication of programmatic failure to find ongoing low-level transmission in a community. However, it should also be noted that the previously determined specificity limits of this serological assay were 96–98%, such that the 3·5% of 1–9 year old samples testing positive may be false positives[11].

Because data were collected from a single community and enrolment was lower than anticipated (primarily due to lack of availability of participants at the time of enrolment, as many adults were working outside of the community at the time of the study), additional studies in post-MDA settings will be needed to confirm the generalizability of our data. While the overall study enrolment was 58.1% of the total population, enrolment of 1–9 year olds, the key WHO indicator age group, was approximately 72.9% (extrapolated from 2010 census data). Without baseline serology data, the inferences we can make about antibody status before MDA, the longevity of antibodies, and how antibody titers change over time in relation to one another are restricted, although our application of catalytic modelling overcomes some of these limitations. While comparing baseline to post-MDA antibody levels would be optimal, programs using serological tests as monitoring tools for intervention impact would need to do so in populations from whom baseline serological data will be absent. Antibody responses will therefore be most useful as surveillance tools by focusing analyses on children born after initiation or cessation of interventions. The data presented in the current study show the power of antibody-based surveillance in children born after cessation of an MDA program, data supported by the historical documentation of interruption of ocular *Ct* transmission in this community.

Antibody responses represent exposure to infection and, when integrated with age, represent exposure over time; this can be done simply by applying a catalytic conversion model. SCR has been used widely in a range of infectious diseases [28, 29], most recently and extensively for malaria, for which SCR has been shown to correlate with the force of infection [15, 30, 31]. Fitting models with two SCRs enabled the measurement of changes in force of infection. SCR suggests a 10-fold decrease in the force of infection from approximately 5% seroconversion in the population per year prior to MDA, to approximately 0.5% after MDA, which closely approximates the 0% ocular infection prevalence seen in this study. Catalytic models can be refined by using serological data from multiple settings, pre- and post-MDA, to further validate the use of serological testing for programs.

Serological tests for measuring antibodies in children may represent the best option for monitoring transmission because of the potential for greater sensitivity as population-based markers of exposure. Additionally, they provide an objective marker, relatively free of observer bias (unlike examination for clinical signs), and are likely to be lower in cost than NAATs and provide data on cumulative exposure to the bacterium. Programmatically, such an assay could be used in the same way that antigen detection assays are used in surveillance for lymphatic filariasis elimination programs, and seroprevalence has been proposed for malaria control and elimination programs [30, 32]; that is, to document reductions in the force of transmission. With the recent increased emphasis on a more horizontal approach to disease control, given similarities in control methods (particularly periodic MDA) and the geographical overlap between trachoma and other NTDs, integration across NTD programs is the next step [33]. This will provide economic and pragmatic benefits, as a multiplexed serological tool has the potential to map, monitor and evaluate several diseases simultaneously, facilitating efforts to achieve long-term elimination goals.

## Supporting Information

S1 Checklist STROBE Statement—Checklist of items that should be included in reports of *cohort studies* included. (DOC)

S1 Table Reproducibility of pgp3 and CT694 serology. Six of seven samples testing positive to either Ct antigen were re-analyzed for antibody-positivity approximately one year after the initial assay. Samples were analyzed using microbead that had been coupled pgp3 or CT694 at a later date than the original test. The cutoffs for the separate bead sets are indicated in row 3. Re-testing of samples repeated the earlier result.
(DOCX)
