# Supplementary material for: Serology for Trachoma Surveillance after Cessation of Mass Drug Administration
Source: PLoS Negl Trop Dis. 2015 Feb 25;9(2):e0003555. doi: 10.1371/journal.pntd.0003555 (PMC4340913; doi:10.1371/journal.pntd.0003555)
Supplement: S1 Table — Six of seven samples testing positive to either Ct antigen were re-analyzed for antibody-positivity approximately one year after the initial assay. Samples were analyzed using microbead that had been coupled pgp3 or CT694 at a later date than the original test. The cutoffs for the separate bead sets are indicated in row 3. Re-testing of samples repeated the earlier result. (DOCX) [file pntd.0003555.s002.docx]

|  | **MFI-BG pgp3** | | **MFI-BG CT694** | |
| --- | --- | --- | --- | --- |
|  | Run 1 | Run 2 | Run 1 | Run 2 |
| Cutoff: | 1024 | 818 | 232 | 150 |
| Sample 1 | 1779 | 935 | 15 | 11 |
| Sample 2 | 23066 | 23832 | 679 | 572 |
| Sample 3 | 5661 | 3122 | 51 | 17 |
| Sample 4 | 2734 | 1511 | 10 | 7 |
| Sample 5 | 2381 | 1913 | 25 | 17 |
| Sample 6 | 13157 | 10661 | 413 | 242 |
